# Paediatric Formulation: Design and Development

**DOI:** 10.3390/ijms21197118

**Published:** 2020-09-27

**Authors:** Antonio Lopalco, Nunzio Denora

**Affiliations:** Department of Pharmacy-Pharmaceutical Sciences, University of Bari Aldo Moro, 70125 Bari, Italy

**Keywords:** formulation, development, design, children, paediatric, age-related, palatable, taste-masking, acceptable, excipient

## Abstract

The development of medicines designed for children can be challenging since this distinct patient population requires specific needs. A formulation designed for paediatric patients must consider the following aspects: patient population variability; dose flexibility; route of administration; patient compliance; drug and excipient tolerability. The purpose of this Special Issue entitled “Paediatric Formulation: Design and Development” is to provide an update on both state-of-the-art methodology and operational challenges in the design and development of paediatric formulations. It aims at re-evaluating what is needed for more progress in the design and development of age-appropriate treatments for paediatric diseases, focusing on: formulation development; drug delivery design; efficacy, safety, and tolerability of drugs and excipients. This editorial, briefly, summarizes the objects of nine original research and review papers published in this Special Issue.

## 1. Introduction

When designing and developing paediatric medicines, the route of administration, dosage form, and dose of the active ingredient (API) are decided on the basis of the expected duration of the therapy, the disease affecting a patient and his/her age, size, physio-pathological condition, API organoleptic and physicochemical properties (taste, aqueous solubility), its pharmacokinetic and pharmacodynamic properties, and stability during manufacture, storage, and use of the chosen formulation [1,2,3,4].

The paediatric population is a heterogeneous group ranging from preterm newborn infants to adolescents with wide physiological and developmental differences regarding organ and skin maturation, metabolism, and other factors that impact on the pharmacokinetics and pharmacodynamics of a drug [5].

It is a challenge to formulate one dosage form appropriate for this heterogeneous group of the human population. The goal should be to safely cover as wide an age range as possible with one specific dosage form. The guiding principle for choosing paediatric formulations should be the equilibrium of risks and benefits, taking into account the specific needs of these vulnerable patients. Key aspects in paediatric formulations involve the design and development of novel dosage forms, which should be adjustable for age and size, acceptable and palatable, easy to administer, and, at the same time, safer and effective [2,3,4].

Current use of medicines for paediatric patients reflects the full range of dosage forms and routes of administration used for adult medicines. There is, however, limited information on the acceptability of different paediatric dosage forms in relation to age and therapeutic needs and on the safety of excipients in relation to the development of the child [2,3,4,6,7].

Since its establishment, the biopharmaceutical classification system (BCS) has facilitated the development of oral drug formulations designed for adult patients. Theoretically, the BCS tenets could be applied also to paediatrics. Children’s peculiarities and physiological differences from adults justify the need for a specific paediatric biopharmaceutics classification system (PBCS). Several scientific works attempted to provisionally classify oral drugs listed on the latest World Health Organization’s Essential Medicines List for Children into age appropriate BCS. Validating a PBCS would provide a valuable tool to apply in specific paediatric formulation design by reducing time and costs and avoiding unnecessary paediatric experiments restricted by ethical reasons. Additionally, PBCS could minimize the associated risks to the use of adult medicines on pharmaceutical compound formulations for children. Moreover, developing a PBCS classification might be helpful in the process of harmonizing extemporaneous oral formulations in the hospital setting [8,9].

As a result of the great interest in developing age-appropriate dosage forms for children, this Special Issue, entitled “Paediatric Formulation: Design and Development”, was programmed to highlight the need of formulating safer and effective medicines for paediatric patients. A total of nine papers were accepted for publication in this issue: four reviews and five primary data manuscripts, focusing on (1) the design, characterization, and safety evaluation of orodispersable formulations for paediatric tuberculosis pharmacotherapy; (2) development and palatability assessment of ritonavir powder for the paediatric population; (3) development of new praziquantel paediatric formulations in schistosomiasis treatment; (4) tridimensional retinoblastoma cultures as a vitreous seeds model for live-cell imaging of chemotherapy penetration; (5) preparation and characterization of dasatinib/cyclodextrin complex for the potential treatment of paediatric neuromuscular disorders; (6) efficacy, safety, and tolerability of tyrosine kinase inhibitors in the treatment of paediatric chronic myeloid leukaemia; (7) technologies and modern approaches to modify drug release in paediatric dosage forms; (8) challenges in formulating neonatal medicines; (9) efficacy, safety, and tolerability of anti-interleukin-1 (anti-IL-1) treatment in paediatric autoinflammatory diseases.

## 2. Articles in This Special Issue

Palatable orodispersible film formulations are ideal for patients with swallowing difficulties such as babies and children because they are stable and dissolve rapidly within the oral cavity in the presence of saliva, without the need to chew or drink water. In this Special Issue, Matawo and co-workers [10] investigated the preparation, optimization, and evaluation of a co-polymeric orodispersible pharmaceutical formulation containing pyrazinamide, a model first line antitubercular agent suitable for use in actively infected children. Organoleptic and cell toxicity studies presented the formulation as palatable, easy-to-handle, and biocompatible under applied test conditions. The orodispersible dosage form developed could potentially ease some of the challenges associated with the safe administration of tuberculosis antibiotics in children to aid desirable pharmacotherapeutic outcomes. Moreover, the authors suggested that the carrier matrix designed in this work might be used as is or even modified to accommodate and safely increase the release and/or absorption of other APIs for use in paediatric patients.

In this Special Issue, Morris and colleagues [11] presented an acceptable and age-appropriate dosage form formulation of the inhibitor of human immunodeficiency virus protease, ritonavir, for paediatric patients or patients who might have difficulties in swallowing a tablet. Norvir^®^ oral powder (NOP) was developed to replace the oral solution, which provided reasonable bioavailability but exhibited taste-masking challenges and required the use of solvents with potential paediatric toxicity. In this study, the authors provided an overview of the development of NOP and palatability assessment strategy. In summary, NOP provided dose flexibility, enhanced stability, eliminated solvents, and maintained consistent bioavailability, with reduced bitterness and improved palatability via administration with common food products.

Here, Albertini and co-workers [12] evaluated the association of mechanochemical activation and spray congealing technology for developing a child-friendly praziquantel (PZQ) dosage form, with better product handling and biopharmaceutical properties, compared to mechanochemical activation materials. PZQ is the first line drug for the treatment of schistosome infections and is included in the WHO Model List of Essential Medicines for Children. In this study, they demonstrated that the approach consisting of the association of spray congealing with mechanochemical activation grinding, in the absence of polymeric excipients, was the most favourable, thus, it could be a promising method for designing a new PZQ formulation and a valid option for enhancing the performance of this antischistosomal drug, possibly permitting a significant reduction in therapeutic dose and minimizing the use of excipients in paediatric formulations.

Retinoblastoma is the most common intraocular tumour of childhood [13,14]. This tumour is highly curable if diagnosed in the early stages. Preclinical models could aid in understanding retinoblastoma vitreous seeds behaviour, drug penetration, and response to chemotherapy to optimize patient treatment. Winter et al. [15] described, in this Special Issue, the development of a novel tridimensional in vitro model of retinoblastoma vitreous seeds to assess chemotherapy penetration by means of live-cell imaging. Under the cell culture conditions used, these cells grew as tumourspheres with the ability to interact in a 3D structure. The results showed that tumourspheres provided a valuable model to study in vitro drug penetration and might be useful to optimize drug therapy and improve the efficacy of the treatment in paediatric patients affected by retinoblastoma.

For the first time, in this issue, a new inclusion complex of dasatinib, the first-choice oral drug in the treatment of chronic myeloid leukaemia and the excipient hydroxypropyl-beta-cyclodextrin (HP-β-CD), was described and fully characterized by Cutrignelli and co-workers [16] for the potential treatment of paediatric neuromuscular disorders. The strategy of complexation with HP-β-CD could allow the development of a paediatric oral liquid formulation of the poor water soluble dasatinib, which could be a valid alternative to the one currently present on the market that is solid. Moreover, considering that HP-β-CD is Food and Drug Administration approved for parenteral formulations, the dasatinib/HP-β-CD inclusion complex could also be an interesting tool for the administration of dasatinib by this route.

Recently, tyrosine kinase inhibitors imatinib, dasatinib, and nilotinib have been approved for the treatment of chronic myeloid leukaemia in children, even though the studies that were concerned with efficacy and safety toward this population are still awaiting defined and more accurate data [17,18,19]. In this scenario, here, Carofiglio and colleagues [20] published a review article pointing out the importance of prospectively validating data extrapolated from adult studies to set a standard therapeutic management for paediatric chronic myeloid leukaemia by employing appropriate formulations on the basis of paediatric clinical trials, which allow a careful monitoring of tyrosine kinase inhibitor-induced adverse effects, especially in growing children exposed to long-term therapy. Limited experience with very young children, the transition of teenagers to adult medicine, and the goal of achieving treatment-free remission for this rare leukaemia are more significant obstacles that require further clinical investigations. Finally, they concluded that in order to carry out a possible and viable therapy with these new anticancer drugs in the paediatric age, a key role is played by developing appropriate formulations specifically customized toward this kind of patient, since these drugs are often available in solid dosage forms, which are difficult to administer in children.

In pharmaceutical technology, the paediatric population still represents the greatest challenge in terms of developing flexible and appropriate drug dosage forms. In their review article published in this issue, Trofimiuk and colleagues [21] elucidated how to modify drug release in paediatric oral dosage forms, discuss the already accessible technologies, and to introduce novel approaches of manufacturing with regard to the paediatric population. Key aspects in modern formulations involve the development of novel formulations when considering chronic diseases that affect children and minimizing the dose frequency. Simultaneously, safety of excipients and child’s acceptability should be kept in mind. The limited number of modified release formulations already present on the market arises from the high cost of technologies and lack of relevant clinical trials in the paediatric population. The authors suggested that new regulations and additional funding opportunities, as well as innovative collaborative research initiatives, should be constantly developed.

In this Special Issue, O’Brien at al. [22] published a review that offered insight into those challenges posed by the formulation of medicinal products for neonatal patients in order to support the development of clinically valid products. Neonates cannot be classified as small children when it comes to medicinal products and their formulation development. To design and develop an appropriate formulation for neonates, it is important to understand their physio-pathological status and development as well as route and methods of drug administration. The paper highlighted that a good understanding of the various constraints that could limit the development of an appropriate paediatric formulation would allow the formulator to provide for neonates whilst having due regard for the needs of the older population. If the neonate is considered early in the formulation design process, some delays in clinical trials in this population might be avoided.

The safety profile of treatment is of paramount importance when children with autoinflammatory disorders are managed, since it may affect adherence to treatment and overall clinical efficacy [23,24,25]. In this regard, in a review paper published in this issue, Bettiol and colleagues [26] aimed at providing current findings on the efficacy, safety, and tolerability of anti-IL-1 agents anakinra and canakinumab in multifactorial autoinflammatory diseases, focusing on the paediatric setting. Recent evidence from both observational studies and clinical trials widely documented the efficacy of IL-1 blocked in the main autoinflammatory diseases, also enlightening a good safety profile with few worries with regard to tolerability. In particular, the observed major side effects of anakinra were skin reactions at the injection-site. These reactions might become so unbearable for paediatric patients that treatment withdrawal might be required. In this regard, convincing young patients to continue therapy could be challenging. Reactions could be mitigated by the application of topical cortisone creams. On the contrary, the overall safety of canakinumab showed an exceptional tolerability, as pointed out by both very few discontinuation rates and injection-site reactions. However, a slightly increased rate of non-serious infections involving the upper respiratory tract was observed. Although these two anti-IL-1 agents currently represent the most effective treatments available in these diseases, and a promising therapeutic tool for managing refractory Kawasaki disease, the development of innovative dosage forms which further reduce side effects in paediatric sceneries is expected.

## 3. Conclusions

The high number of articles published in this Special Issue entitled “Paediatric Formulation: Design and Development” highlights the significant amount of research being conducted on the development of medicines designed for paediatric patients.

Formulating an appropriate dosage form is a challenge when considering chronic diseases that affect children and minimizing the dose frequency. Key aspects in paediatric formulations involve the design and development of novel dosage forms, which should be adjustable for age, palatable, easy to administer, and, at the same time, safer and effective.
